# The Emotional Status, Attitudes in Decision-Making Process, and Their Impact on Surgical Choices in Korean Breast Cancer Patients

**DOI:** 10.1155/2021/6636986

**Published:** 2021-03-12

**Authors:** Sook Young Jeon, Kyoung-Eun Kim, Eun-Kyu Kim, Hyunhee Han, Han-Byoel Lee, Wonshik Han, Dong-Young Noh, Hyeong-Gon Moon

**Affiliations:** ^1^Department of Surgery, Seoul National University College of Medicine, Seoul, Republic of Korea; ^2^Pusan National University Hospital, Busan Cancer Center, Pusan, Republic of Korea; ^3^Department of Surgery, Seoul National University College of Medicine, Seongnam, Republic of Korea; ^4^Cancer Research Institute, Seoul National University, Seoul, Republic of Korea

## Abstract

**Purpose:**

We examined the incidence of emotional distress in women with newly diagnosed breast cancer to determine whether the degree of emotional distress affected their choice of breast-conserving surgery (BCS) or mastectomy and evaluated how the patient's preferred role in decision-making influenced her choice of surgical method.

**Methods:**

This prospective study included 85 patients newly diagnosed with in situ or invasive breast cancer eligible for BCS. Their degree of depression/anxiety and attitude toward the decision-making process were measured using the Hospital Anxiety and Depression Scale (HADS) and Control Preference Scale (CPS), respectively. After receiving information on both surgical methods, the patients indicated their preferred surgical method and completed the CPS at their initial and second visits before surgery.

**Results:**

After the diagnosis of breast cancer, 75.3% of patients showed abnormal or borderline HADS scores for depression and 41.2% for anxiety. Patients with borderline or abnormal degrees of depression were more likely to have coexisting abnormal degrees of anxiety (*p* < 0.001). However, the presence of depression or anxiety was not associated with patients' surgical choices (*p*=0.394 and 0.530, respectively). Patients who preferred a more active role in the decision-making process were more likely to choose mastectomy over BCS, while those who were passive or collaborative chose BCS more frequently (*p*=0.001).

**Conclusion:**

Although many patients with newly diagnosed breast cancer experience depression and anxiety before surgery, these do not affect the choice of surgical method; however, their attitudes toward the decision-making process do.

## 1. Introduction

Breast-conserving surgery (BCS) is a well-established treatment option for early breast cancer patients with a comparable survival to that of mastectomy [1, 2]. Recent large population-based retrospective studies have suggested that the survival outcomes of BCS are similar or superior to those of mastectomy [3–8]. While there are expert opinions that stress the benefit of breast conservation over mastectomy based on the potential benefit of survival observed in the large retrospective studies [5, 9], most patients with early breast cancer are still offered with both surgical options [10]. However, patients with early breast cancer often face a complex and difficult decision-making process when choosing their own surgery type at the time of diagnosis. Patients must weigh their concerns about radiation and recurrence against the detrimental emotional effect of removing the whole breast.

The decision-making process regarding the type of breast cancer surgery is affected by a wide array of factors, including the clinicopathological factors of the tumor, attitudes of the individual patients, and advice from the treating surgeon. According to the recent systematic review by Gu et al. [11], patients' individual beliefs, including peace of mind or fear of local recurrence, play a significant role in deciding the type of breast surgery, as do their demographic characteristics such as age, education, socioeconomic status, and race [11].

The decision for breast conservation or mastectomy is often further complicated by the emotional stress that newly diagnosed breast cancer patients undergo. Being diagnosed with breast cancer is experienced as a major challenge and constitutes an important stressor in a women's life that may cause emotional distress, particularly anxiety and depression [12], whose prevalence among patients diagnosed with breast cancer is reported to exceed 30% [13, 14]. However, it is not clear how these emotional disturbances affect patients' choice of breast surgery.

In the present prospective trial, we investigated the incidence of emotional distress in Korean women with newly diagnosed breast malignancies and assessed their attitude toward the treatment decision-making process during the discussion of different types of breast cancer surgery to determine how their degree of emotional distress and attitude toward the decision-making process affected the final choice of surgery type.

## 2. Materials and Methods

### 2.1. Patient Population

Between August and December 2018, we invited a total of 107 patients to participate in our study. The inclusion criteria were patients newly diagnosed with invasive breast carcinoma or in situ carcinoma, older than 20 years of age, not candidates for neoadjuvant systemic therapy, and eligible for breast conservation surgery based on their clinical tumor size at initial presentation. This prospective study was approved by the Institutional Review Board (IRB no. 1807-054-957) and was conducted according to the Declaration of Helsinki. This trial was registered on the CRIS website (https://cris.nih.go.kr) with the identification number KCT0004736. All participants provided written informed consent.

### 2.2. Study Design and Questionnaire

**Figure 1:**
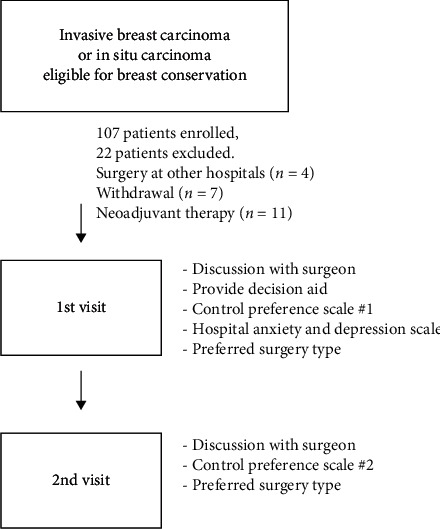
Enrollment and study design.

During their first visit to our breast cancer clinic, the newly diagnosed breast cancer patients who met the inclusion criteria were asked to participate in our study. The patients were provided with information regarding the advantages and disadvantages of both breast conservation and mastectomy, and decision aids were provided. We modified the decision aid reported by Alam et al. [15] and translated into Korean. The re-operation rate after breast cancer surgery described in Alam et al.' decision aid was 20%, which was reduced to 10% in our decision aid based on a review of the institution's surgical outcome data. The decision aid used in our study is shown in Figure 1. Immediately after the initial visit, the patients were surveyed using the Control Preference Scale (CPS) and Hospital Anxiety and Depression Scale (HADS) and were asked their preferred surgical method. The CPS measures the degree of control that an individual wants to assume on medical decision-making [16] and classifies patients into three groups: patient-controlled, jointly controlled, and provider-controlled. The HADS comprises 14 items developed to measure the levels of anxiety and depression in patients [17].

On the second visit, patients had additional discussion with their surgeons based on the results of imaging studies. During the second visit, patients were asked for their preferred surgical method and were surveyed for the second CPS questionnaire.

### 2.3. Statistical Analysis

The degree of patients' depression and anxiety was measured using the Depression Subscale (HADS-D) and Anxiety subscale (HADS-A) of the HADS questionnaire. Patients were classified into three groups based on their subscale scores (0–7 normal, 8–10 borderline, and 11–21 abnormal) [17]. Information on the clinical and pathologic characteristics of patients was obtained by reviewing electronic medical records. Patients were classified according to the CPS and HADS survey results, whose associations with surgical choices were analyzed using the chi-square test. Continuous variables were compared using Student's *t-test* or ANOVA.

## 3. Results

### 3.1. Characteristics of the Patients

A total of 85 patients diagnosed with ductal carcinoma in situ or invasive breast cancer who were eligible for breast conservation between August 8 and December 31, 2018, participated in this study. The baseline clinical information is listed in Table 1. As the inclusion criteria included eligibility for breast conservation, most patients had been diagnosed with small tumors with a median tumor size of 1.9 cm.

### 3.2. Emotional Distress and Surgical Choices

The degree of depression and anxiety was measured using the HADS questionnaire [17] on their first visit to our hospital after their diagnoses of breast malignancy. Of these 85 patients, the majority showed abnormal (*n* = 50, 58.8%) or borderline (*n* = 14, 16.5%) scores for depression, while only 21 patients (24.7%) displayed normal ranges. Regarding anxiety, 13 (15.3%) and 22 patients (25.9%) showed abnormal and borderline HADS scores, respectively. Patients with borderline or abnormal degrees of depression after the diagnosis of breast malignancy were more likely to have coexisting abnormal degrees of anxiety (*p* < 0.001). Although most demographic or clinical features were not associated with emotional disturbance (Table 2), patients diagnosed with invasive breast cancer had a significantly higher incidence of depression than those with in situ tumors (*p*=0.031).

Next, we investigated whether the levels of depression or anxiety in these newly diagnosed patients affected their choice of type of breast surgery. After providing information on both mastectomy and breast conservation using decision aids, each patient's preference for surgical method was recorded on the initial and second visit, when the surgical methods were discussed again and finalized. Seventeen patients (20%) had difficulty in deciding their surgical choice at the first visit, which decreased to six (7.1%) at the second visit. The presence of borderline or abnormal degrees of depression or anxiety was not significantly associated with the patients' surgical choices (*p*=0.394 and 0.530, respectively; Table 3). Mastectomy was chosen by 5 of the 21 (23.8%) and 13 of the 64 (20.3%) patients in the normal and the borderline-abnormal depression groups, respectively, and by 11 of the 50 (22.0%) and 7 of the 35 (20%) patients in the normal group and the borderline-abnormal anxiety groups, respectively.

### 3.3. Preferred Role in the Treatment Decision and Surgical Choice

We also assessed each patient's control preference in the medical decision-making process during their selection of breast surgery type (mastectomy or breast conservation). Patients were asked about their preferred role in the decision-making process using the modified CPS, as suggested by Chiu et al. [18], before their first and second visits before the surgery. The discussion between the surgeon and the patient using the decision aid significantly changed the patients' attitudes toward surgical decision-making (*p*=0.003, Table 4). Before the first visit, 32 patients (37.6%) were classified as passive (provider-controlled) and only two patients as active (patient-controlled). However, at the second visit, there was a 29% reduction in the passive group and a four-fold increase in the active group (23 in the passive group and 8 in the active group at the second visit).

Furthermore, we observed that the patients' preferred role in the decision-making process was significantly associated with their choice of breast surgery. Patients who preferred a more active role in the decision-making process were more likely to choose mastectomy over breast conservation, while patients who were either passive or collaborative chose breast conservation more frequently (*p*=0.001, Table 4). The most common reason for choosing mastectomy as their preferred surgery was the fear of tumor recurrence, followed by concerns about possible re-operation.

## 4. Discussion

Being diagnosed with breast cancer constitutes a major challenge and an important stressor in a woman's life that may cause various emotional distresses, particularly anxiety and depression [12]. Causes of psychological distress are treatment-related distress, worries regarding fear of death and recurrence, and altered body image, sexuality, and attractiveness [19, 20]. Studies have shown that the prevalence of anxiety and depression among breast cancer patients is high, exceeding 30% [13, 14]. Additionally, Kim et al. have shown that the incidence of hospital visits for depression is significantly higher for several years after the initial surgery in Korean breast cancer patients [21]. In the present study, we observed a high incidence of patients suffering from anxiety and depression after their diagnosis of breast malignancies. More than 70% and 40% of newly diagnosed patients displayed abnormal ranges of depression and anxiety, respectively. Our observations fall within the previously reported incidences of emotional distress in newly diagnosed breast cancer patients [20, 22–25].

Studies have thoroughly investigated whether the breast surgery type significantly influences patients' emotional status, and although some reported a modest advantage for breast conservation, most found that mastectomy and breast conservation have similar effects on emotional outcomes in breast cancer patients [26–28]. In this study, we asked whether the presence preoperative emotional distress can affect patients' decisions regarding surgery types since the fear of recurrence is a major factor in determining mastectomy. However, our analysis shows that the degree of preoperative emotional distress in the newly diagnosed breast cancer patients is not associated with patients' choice in surgery type.

Interestingly, we observed that patients' attitudes in the decision-making process were significantly associated with their choice of mastectomy in the present study. Patients in the “active” decision-making group (more patient-controlled) were more likely to choose mastectomy than other groups. Hawley et al. [29] also reported that greater patient involvement in decision-making was associated with a higher incidence of receiving mastectomy in their study involving 1,651 patients. However, their study used data obtained from a mail survey of breast cancer patients identified by the SEER reports. Although they limited their analytic samples to recently diagnosed cases, their study was not free from recall bias. In the present study, we obtained all survey information during the preoperative period, so the patients' attitudes were more accurately reflected in the survey results.

Our study has several limitations. First, the decision-making process of breast cancer patients is a complex process affected by multiple factors. Molenaar et al. [30] reported that, in their prospective trial involving 180 early breast cancer patients, the patients' choice for breast conservation or mastectomy was influenced by the treatment preference of the surgeons and their concerns about the loss of their breast and local recurrence. In the present study, the treating surgeons provided only the pros and cons of each surgery type based on the information in the decision aid, and the patients were encouraged to make their own decisions. The surgeon's advice was given only to individual patients who could not decide on the surgery type through the two discussion sessions. Second, our observations need further validation in a larger cohort, given the small number of patients enrolled in the present study.

In conclusion, this prospective trial demonstrates that there is a high incidence of emotional distress in newly diagnosed Korean breast cancer patients. Emotional distress did not influence the decision-making process on breast surgery type; however, their attitude toward the decision-making process showed a significant association with their preferred surgery type. Patients with more active involvement in the decision-making process were more likely to undergo mastectomy over breast conservation.

## Figures and Tables

**Table 1:** Clinicopathologic characteristics of study subjects.

Characteristics	Median ± SD or number	Range or %
Age	52.9 ± 11.1	(25, 80)
BMI	23.3 ± 3.7	(16.2, 35.2)
Education status		
College or university education	32	41
High school education	46	59

Occupation		
No	44	54.3
Yes	37	45.7

Marital status		
Unmarried	14	16.7
Currently married	70	83.3

FHx of breast cancer		
No	78	91.8
Yes	7	8.2

FHx of other cancers		
No	82	96.5
Yes	3	3.5

Menopausal status		
Premenopause	43	50.6
Postmenopause	42	49.4
Tumor size	1.9 ± 1.3	(0.5, 7)

*T* stage		
Tis	19	22.6
*T*1	42	50
*T*2	23	27.3

*N* stage		
*N*0	57	86.4
*N*1	8	12.1
*N*2	1	1.5

Diagnosis		
Invasive	65	76.5
In situ	20	23.5

**Table 2:** Characteristics according to degree of depression and anxiety.

	Depression			*p* value	Anxiety			*p* value
	Normal	Borderline	Abnormal		Normal	Borderline	Abnormal	
Age	55.1 ± 11.3	52.7 ± 13.7	52.1 ± 10.3	0.583	54.2 ± 11.3	51.5 ± 10.5	50.9 ± 11.4	0.5
BMI	24.5 ± 4.3	23.1 ± 3.9	22.9 ± 3.3	0.228	23.7 ± 3.7	23.2 ± 4.1	22.0 ± 3.1	0.372
Education status								
High school education	11 (55.0)	6 (50.0)	29 (63.0)	0.686	26 (56.5)	11 (23.9)	9 (19.6)	0.522
College or university education	9 (45.0)	6 (50.0)	17 (37.0)		21 (65.6)	8 (25.0)	3 (9.45)	

Occupation								
No	11 (55.0)	9 (64.3)	24 (51.1)	0.71	26 (59.1)	10 (22.7)	8 (18.2)	0.732
Yes	9 (45.0)	5 (35.7)	23 (48.9)		23 (62.2)	10 (27.0)	4 (10.8)	

Menopausal status								
Premenopause	8 (38.1)	7 (50.0)	28 (56.0)	0.369	21 (42.0)	13 (59.1)	9 (69.2)	0.158
Postmenopause	13 (61.9)	7 (50.0)	22 (44.0)		29 (58.0)	9 (40.9)	4 (30.8)	
Tumor size	1.8 ± 1.3	2.3 ± 2.1	1.9 ± 1.0	0.54	1.8 ± 1.2	2.2 ± 1.4	2.1 ± 1.3	0.435

*T* stage								
Tis	7 (33.3)	6 (42.9)	6 (12.0)	0.157	13 (26.0)	4 (18.2)	2 (15.4)	0.164
*T*1	9 (42.9)	5 (35.7)	28 (56.0)		24 (48.0)	8 (36.4)	10 (76.9)	
*T*2	5 (23.8)	3 (21.4)	15 (30.0)		12 (24.0)	10 (45.5)	1 (7.7)	

Diagnosis								
Invasive	14 (66.7)	8 (57.1)	43 (86.0)	0.031	36 (72.0)	18 (81.8)	11 (84.6)	0.589
In situ	7 (33.3)	6 (42.9)	7 (14.0)		14 (28.0)	4 (18.2)	2 (15.4)	

Anxiety								
Normal	21 (100)	12 (85.7)	17 (34)	<0.001				
Borderline	0	2 (14.3)	20 (40.0)					
Abnormal	0	0	13 (26.0)					

Values are presented as number of patients (%). HADS score: 0–7, normal; 8–10, borderline; 11–21, abnormal.

**Table 3:** Association between emotional status and surgical choice.

	Depression			*p* value	Anxiety			*p* value
	Normal	Borderline	Abnormal		Normal	Borderline	Abnormal	
Breast conservation	13 (61.9)	9 (64.3)	39 (78.0)	0.394	35 (70.0)	17 (77.3)	9 (69.2)	0.53
Mastectomy	5 (23.8)	4 (28.6)	9 (18.0)		11 (22.0)	5 (22.7)	2 (15.4)	
No decision	3 (14.3)	1 (7.1)	2 (4.0)		4 (8.0)	0 (0)	2 (15.4)	

Values are presented as number of patients (%).

**Table 4:** Association between the Control Preference Scale and surgical choice.

	1st visit			*p* value	2nd visit			*p* value
	Active	Collaborative	Passive		Active	Collaborative	Passive	
Distribution	2 (2.4)	51 (60)	32 (37.6)		8 (9.4)	54 (63.5)	23 (27.1)	0.003

Surgical choice								
Breast conservation	2 (100)	34 (66.7)	21 (65.6)	1.0	2 (25)	43 (79.6)	16 (69.6)	0.001
Mastectomy	0	7 (13.7)	4 (12.5)		6 (75)	9 (16.7)	3 (13.0)	
Not decided	0	10 (19.6)	7 (21.9)		0	2 (3.7)	4 (17.4)	

Values are presented as number of patients (%).

## Data Availability

The data used to support the findings of this study are available on request to the corresponding author.
